# Effects of Shexiang Baoxin pill combined with exercise rehabilitation training on long-term prognosis of CTO-PCI patients: study protocol for randomized controlled trial

**DOI:** 10.3389/fmed.2025.1668432

**Published:** 2025-10-23

**Authors:** Ziye Liu, Shuo Liang, Xiaoping Guo, Yuying Zhao

**Affiliations:** ^1^Medical Technology College, HeBei Medical University, Shijiazhuang, Hebei, China; ^2^School of Nursing, HeBei University of Chinese Medicine, Shijiazhuang, Hebei, China; ^3^Department of Cardiology, No. 980 Hospital of PLA Joint Logistics Support Force, Shijiazhuang, Hebei, China

**Keywords:** coronary chronic total occlusion (CTO), coronary microvascular dysfunction (CMVD), Shexiang Baoxin pill (SBP), exercise rehabilitation, long-term prognosis

## Abstract

**Background:**

CTO-PCI is generally successful, but 40–55% of patients experience coronary microvascular dysfunction (CMVD) afterward, raising the risk of cardiovascular issues. Shexiang Baoxin Pill (SBP) aids angiogenesis and cardiovascular health, while exercise rehabilitation boosts coronary collateral circulation. However, the combined impact of these treatments on CTO-PCI patients remains under-researched.

**Methods:**

This prospective, single-center, randomized controlled trial involves CTO-PCI patients divided into four groups: Shexiang Baoxin pill, exercise rehabilitation, combined, and control. The exercise protocol includes pre-assessment, a three-phase prescription, CPET-based intensity grading, various exercise methods, remote monitoring, and quality control. The primary outcome is coronary microcirculation assessed by myocardial contrast echocardiography and stress myocardial perfusion imaging 1-year post-surgery. Secondary outcomes include angina incidence, major adverse cardiovascular events (MACE), quality-of-life improvements, and mechanism-related biological indicators, with safety outcomes monitoring liver and kidney function. Data will be analyzed statistically.

**Discussion:**

This study will demonstrate the positive effects of Shexiang Baoxin pill combined with exercise rehabilitation training in improving coronary microcirculatory conditions, reducing major adverse cardiovascular events, and improving quality of life in patients with CTO-PCI. The results of this study will provide new insights into the CTO-PCI patients’ long-term rehabilitation.

**Clinical trial registration:**

ITMCTR2025000965, Effect of Shexiang Baoxin Pill combined with exercise rehabilitation training on the long-term prognosis of patients with CTO-PCI, Registered on 28 February, 2025.

## 1 Background

Coronary chronic total occlusion (CTO) percutaneous coronary intervention (PCI) remains a cornerstone in revascularization strategies, with contemporary studies demonstrating improved procedural success rates exceeding 90% in experienced centers (1). However, long-term outcomes remain concerning, as 40–55% of patients develop coronary microvascular dysfunction (CMVD) post-PCI, significantly increasing risks of recurrent ischemia and major adverse cardiovascular events (MACE). According to a meta-analysis’s subgroup analysis, 36.04% of patients with chronic coronary syndrome (CCS) experience CMVD following PCI (2). About 17.86% of patients with CCS had CTO (3). Numerous studies have established a strong link between CMVD and an increased risk of cardiovascular events (4). For example, in patients with ST-segment elevation myocardial infarction (STEMI), CMVD is common and is closely associated with left ventricular diastolic dysfunction, functional left ventricular remodeling (FLVR), and MACE (5). In obese patients, CMVD not only correlates with an elevated risk of adverse cardiovascular events but also serves as a more robust predictor than body mass index (BMI) (6). This disparity between high procedural success and suboptimal long-term outcomes presents an acute clinical challenge. Although we are proficient in reopening occluded epicardial arteries, protecting the fragile microvasculature from ischemia–reperfusion injury remains a significant hurdle. Addressing this challenge requires further exploration of diagnostic and therapeutic approaches to manage patients at high risk of CMVD-related complications.

Shexiang Baoxin pill (SBP), a representative formulation of traditional Chinese medicine, demonstrates its therapeutic efficacy through the synergistic action of multiple constituents. Artificial musk improves blood circulation and reduces myocardial ischemia. Ginseng boosts qi, modulates the immune system, and supports heart function. Suhexiang aids qi circulation and pain relief, helping alleviate angina symptoms. Calculus Bovis, Bufonis Venenum, and Cinnamomi Cortex clear heat and detoxify, while Borneolum Syntheticum enhances delivery of all components. Recent pharmacological research has substantiated that SBP facilitates the release of nitric oxide by vascular endothelial cells, leading to the dilation of coronary arteries and an increase in coronary blood flow (7). It also inhibits inflammatory cytokines, thereby reducing vascular wall inflammation and stabilizing atherosclerotic plaques. Additionally, the pill promotes angiogenesis, establishing collateral circulation for ischemic myocardium and enhancing myocardial blood supply (8). By improving vascular endothelial function, it aids in the release of vasodilatory substances such as nitric oxide, thereby enhancing blood flow (9). Furthermore, it suppresses vascular wall inflammation by reducing the infiltration of inflammatory cells and the release of harmful mediators, thus slowing the progression of atherosclerosis (10). The pill also encourages therapeutic angiogenesis, stimulating the growth of new blood vessels to support the ischemic myocardium. Its active components, including muscone and ginsenosides, enhance endothelial nitric oxide synthase (eNOS) coupling, reduce nod-like receptor pyrin domain containing 3 (NLRP3) inflammasome activation, and promote VEGF-mediated angiogenesis (11, 12).

Exercise rehabilitation is crucial for managing coronary heart disease by improving coronary artery collateral circulation and microcirculation (13). Regular physical activity strengthens alternative blood flow pathways and enhances oxygen and nutrient delivery to the heart muscle (14). Furthermore, structured exercise rehabilitation has been shown to confer significant microcirculatory benefits through mechanisms such as shear stress-mediated endothelial repair and the activation of the hypoxia-inducible factor 1α (HIF-1α) pathway (15). A meta-analysis of multiple randomized controlled trials has demonstrated that exercise rehabilitation reduces the risk of major cardiovascular events, such as heart attacks and strokes, by 28% in patients with coronary heart disease (16–18). Another study assessed the comparative benefits of cardiac rehabilitation strategies through a Bayesian network meta-analysis, revealing that exercise-only cardiac rehabilitation was associated with decreased cardiovascular mortality and major adverse cardiovascular events (19). Despite these advancements, there is limited research on combining SBP and exercise rehabilitation for those undergoing CTO-PCI. The 2023 ESC guidelines recommend microvascular protection but do not provide specific protocols for integrative approaches. This gap in knowledge hinders the personalized management of high-risk CMVD patients.

The combined use of SBP and exercise rehabilitation offers synergistic benefits, particularly in improving myocardial blood supply. SBP promotes angiogenesis, increasing coronary microvessel number and diameter, while exercise rehabilitation enhances collateral circulation, boosting blood perfusion efficiency in both new and existing microvessels. This integrated approach is more effective in mitigating myocardial ischemia. We suggest that synchronizing SBP with exercise rehabilitation enhances therapeutic outcomes through three aspects: (1) improved coronary microcirculatory function; (2) reduced major adverse cardiovascular events within a year; and (3) enhanced patient quality of life. This study introduces an integrative model for post-PCI microvascular rehabilitation, merging traditional Chinese medicine’s systemic regulation with Western exercise physiology’s biomechanical precision, marking a shift from fragmented approaches. Successful validation could reshape international guidelines, providing the first Class I recommendation for combined pharmaco-mechanical CMVD management.

## 2 Methods

### 2.1 Study design

**Figure 1:**
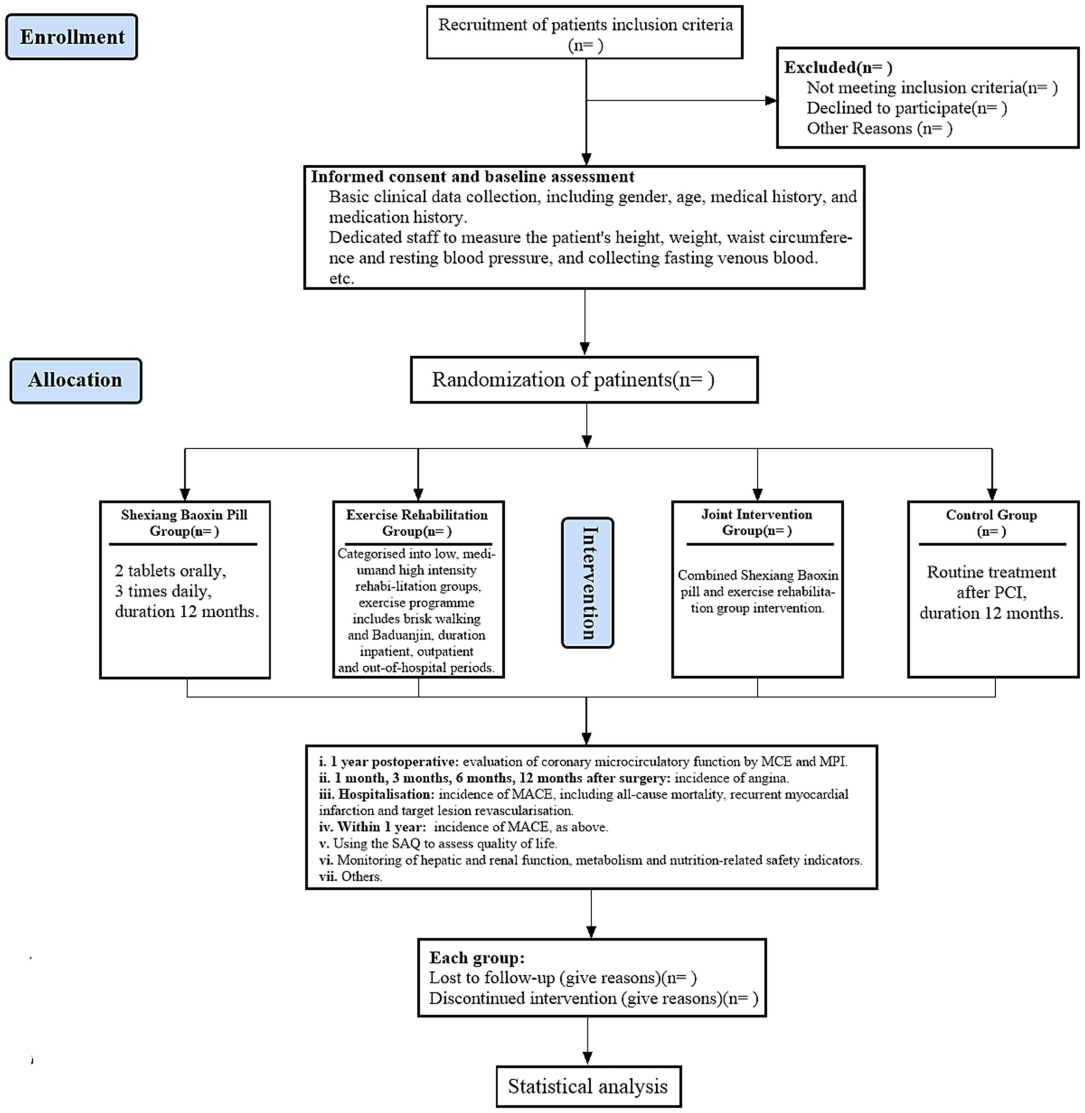
The flowchart of the study process.

This study is a prospective, single-center, randomized controlled clinical trial. Patients with CTO who underwent PCI in the cardiology department of our hospital. Using a computer-generated block randomization sequence (block size = 4), participants were allocated in a 1:1:1:1 ratio to one of four parallel groups: the SBP group, the exercise rehabilitation group, the combined SBP and exercise rehabilitation group, and the control group. Allocation concealment was ensured by a central randomisation module embedded in the Hospital Information System (HIS); the treatment assignment was automatically released only after the participant had been registered and baseline data entry was completed. Figure 1 shows the details of the study process. This study will strictly adhere to the Standard Protocol Items: Recommendations for Interventional Trials (SPIRIT) reporting standard (20).

### 2.2 Study population

#### 2.2.1 Inclusion criteria

Patients aged >18 years old, regardless of gender, diagnosed with CTO through coronary angiography.

Patients with evidence of myocardial ischemia who are planned to undergo or have successfully completed CTO-PCI.

Patients who can understand the purpose of the study, are willing to participate, sign the informed consent form, and are eager to follow up as required.

Patients with instruments who possess devices that measure fundamental physiological indicators (e.g., electronic blood pressure monitors).

#### 2.2.2 Exclusion criteria

Patients with cardiovascular and cerebrovascular accidents (such as acute myocardial infarction, cerebral hemorrhage, and cerebral embolism) within the past 6 months.

Patients with severe heart failure symptoms or left ventricular ejection fraction <40%.

Patients with severe renal insufficiency requiring dialysis.

Patients with bleeding tendency, a history of active peptic ulcer, cerebral hemorrhage or subarachnoid hemorrhage, a history of stroke within the past 6 months, and contraindications to antiplatelet and anticoagulant therapy.

Patients with sick sinus syndrome, second-degree type I or third-degree atrioventricular block.

Unstable angina patients with resting angina attacks within 48 h.

Patients with a life expectancy <12 months.

Patients with significantly limited exercise capacity.

Patients with poor compliance who cannot complete the study as required.

### 2.3 Intervention measures

As an interventional trial, blinding of participants, treating physicians, and researchers directly involved in intervention implementation was not feasible due to the specific nature of the interventions (including oral SBP and exercise rehabilitation training), whose distinct characteristics would render blinding impractical. Importantly, outcome assessors responsible for data collection and statisticians conducting post-data-collection analyses remained blinded to group allocation throughout the study.

If a participant learns their group assignment during rehabilitation exercises, they will be assessed. If this knowledge could influence their behavior, they may be excluded from the study. Additionally, other participants will be monitored for information leaks. If others are affected, measures like re-evaluating data validity or exclusion from the study will be taken.

**Figure 2:**
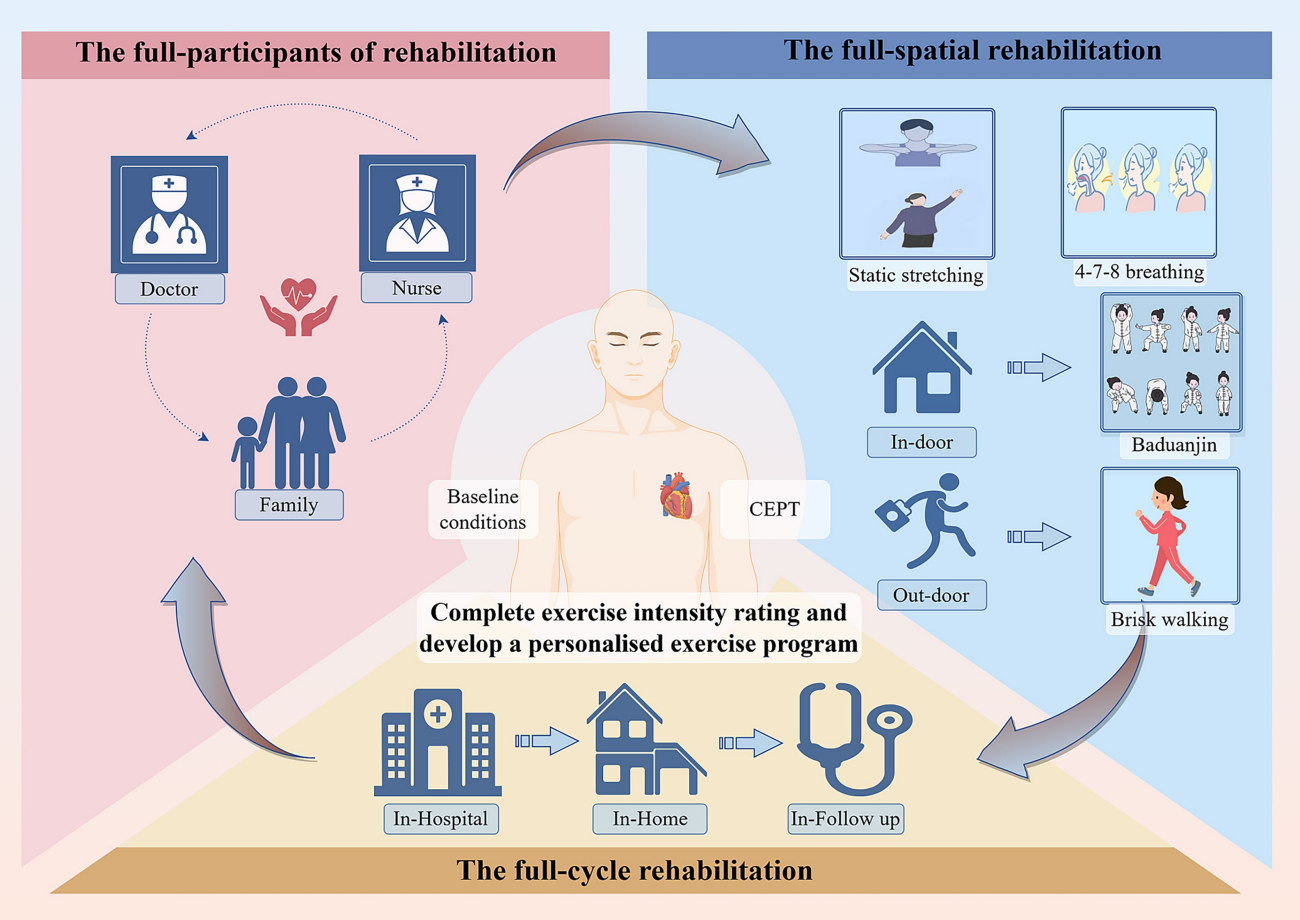
The principles of full-cycle rehabilitation.

**Figure 3:**
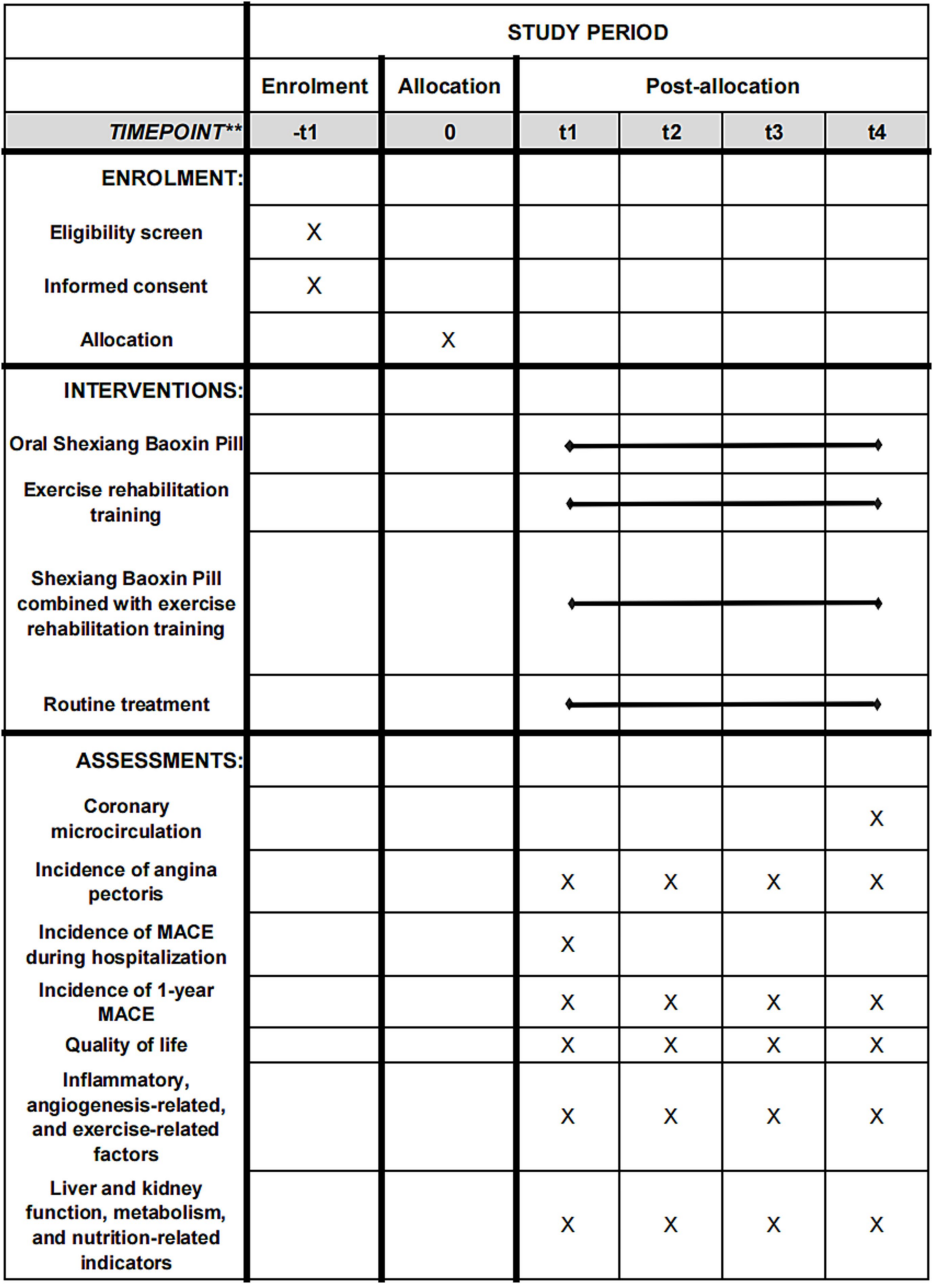
Standard protocol process, schedule covering enrolment, interventions, and assessments (according to SPIRIT).

Tables 1–6 show the details content of the pre-exercise assessment, the three-phase exercise prescription, the exercise intensity grading, the type of exercise, the remote monitoring, and the outpatient period training program, respectively. The principles of full-cycle rehabilitation are shown in Figure 2. The schedule in this study is shown in Figure 3.

**Table 1:** Pre-exercise assessment.

Component	Detailed design
Cardiopulmonary function	CPET to measure VO_2_ peak and anaerobic threshold (AT)
Exercise risk stratification	AACVPR risk stratification criteria (incorporating LVEF, arrhythmia history)
Muscle function evaluation	30-s chair stand test, grip strength measurement
Psychological assessment	Hospital anxiety and depression scale (HADS)
Vascular evaluation	Coronary flow reserve (CFR), index of microcirculatory resistance (IMR, optional)

CPET, cardiopulmonary exercise test; AACVPR, American Association of Cardiovascular and Pulmonary Rehabilitation, LVEF, left ventricular ejection fraction.

**Table 2:** Three-phase exercise prescription.

Phase	Timeframe	Primary goals	Monitoring	Termination Criteria
Phase I	(Inpatient) 2–7 days post-PCI	Prevent deconditioning	Daily assessment	Chest pain > CCS Class II, SpO2 < 90%
Phase II	(Outpatient) 1–6 months post-discharge	Improve aerobic capacity	Weekly onsite + remote	ST depression ≥2 mm, SBP drop >10 mmHg
Phase III	(Home-based) 7–12 months	Long-term maintenance	Monthly follow-up	New-onset VT/AF

CCS, Canadian Cardiovascular Society, VT’AF, ventricular tachycardia/atrial fibrillation.

**Table 3:** Based on CPET exercise intensity grading.

Intensity grading based on CPET	Target parameters	Applicable phases	Patient characteristics
Low intensity	40–50% of HR_peak_, RPE 11–12	Phase I/high-risk patients	LVEF < 45%, NYHA functional class II
Moderate intensity	60–70% of HR_peak_, RPE 13–14	Core of Phase II	No residual ischemia, ≥5 METs
High intensity	80–85% of HR_peak_, RPE 15–16	Optimization of Phase III	Young, low-risk, and well-adapted

RPE, rating of perceived exertion, LVEF, left ventricular ejection fraction NYHA, New York Heart Association; METs, metabolic equivalents.

**Table 4:** Type of exercise training.

Exercise modality	Details
Outdoor Aerobic training	Method: brisk walking Frequency: 3–5 times a week; duration: 30–45 min per session. Progression: increase the duration or intensity by 5% every month, or increase the walking distance and speed gradually according to your own situation.
Indoor Aerobic training	Method: Baduanjin Frequency: 3–4 times a week; duration: 12–26 min per session; take a 2-min break between each set. Precautions: perform the movements standardized, coordinate breathing with the movement rhythm, and avoid overexertion.
Flexibility training	Method: static stretching (30 s per muscle group) Focus: correcting the pectoralis major and minor muscles
Breathing training	Technique: 4–7-8 breathing technique Goal: reduce the oxygen consumption of the respiratory muscles and increase blood oxygen levels

Recorded videos of aerobic, resistance, flexibility, and breathing training will be uploaded to the WeChat for participants’ reference.

**Table 5:** Remote monitoring system.

Methods	Details
Equipment for measuring physiological indicators	Electronic blood pressure monitors
Monitoring platform	Custom APP features: - HR, resting BP, etc. upload - Exercise video upload

HR, heart rate, BP, blood pressure.

**Table 6:** Sample outpatient session (Phase II).

Time	Activity
8:00–8:30	Ambulatory BP monitoring + pre-exercise assessment (Borg scale)
8:30–8:45	Chest expansion and front raise exercise with heavy weights in hand (weight depends on the individual and increases over time)
8:45–9:30	Brisk walking: walk at a moderate speed for 30–45 min. Adjust the speed appropriately during the process to maintain the exercise intensity
9:40–10:10	Baduanjin: practice Baduanjin completely. Perform the movements standardized, coordinate breathing, and do 1–2 rounds, which takes about 12–26 min
10:15–10:35	Breathing exercise: 4–7-8 breathing technique
10:35–10:50	Cool-down + post-exercise ECG monitoring

#### 2.3.1 Shexiang Baoxin pill intervention

Patients in the SBP group will start taking two pills, three times a day orally after surgery for 12 months.

#### 2.3.2 Exercise rehabilitation intervention

Patients in the exercise rehabilitation group will undergo a comprehensive assessment before exercise, including general function assessment, exercise risk assessment, exercise tolerance assessment, and psychological assessment. Then, the exercise intensity will be set according to their individual conditions and cardiopulmonary exercise test results. They will be divided into low-intensity, medium-intensity, and high-intensity rehabilitation groups. The exercise program will include aerobic exercise, resistance exercise, and flexibility exercise. During the hospitalization period (acute phase, phase I), the responsible nurse will guide patients to perform rehabilitation exercises. After discharge, patients will receive early outpatient rehabilitation (stable phase, phase II) and maintenance-phase outpatient rehabilitation. The responsible nurse will conduct weekly telephone follow-ups to monitor and guide patients’ exercise implementation.

#### 2.3.3 Combined intervention

Patients in the SBP combined with exercise rehabilitation group will receive both SBP treatment and exercise rehabilitation training as described above.

#### 2.3.4 Control group

Patients in the control group will receive routine post-PCI treatment.

### 2.4 Data collection and outcome measures

Eligible patients will be informed of the trial, agree to participate, and voluntarily sign the informed consent form. At the first visit, clinicians will collect basic clinical data of patients, including gender, age, medical history, and medication history. The next day, special personnel will measure patients’ height, weight, waist circumference, and resting blood pressure, and collect fasting venous blood for biochemical tests. In the data collection process, participants are assigned ID numbers, which are used as their identifiers in the system to safeguard their real identities. The collected information is stored in a highly secure database with strict access control, accessible only to the study leader and statistical experts. This ensures effective protection of participants’ privacy throughout the trial. Details of the outcome measures are shown in Table 7.

**Table 7:** Details of the outcome measures.

Outcome type	Specific content	Evaluation time	Evaluation method
Primary outcome	Evaluation of coronary microcirculation	Immediately and 1 year after revascularization	Myocardial contrast echocardiography (MCE): MBF; myocardial perfusion imaging (MPI): SDS
Secondary outcomes	Incidence of angina pectoris	1 month, 3 months, 6 months, and 12 months after revascularization	Clinical observation and recording
Secondary outcomes	Incidence of MACE during hospitalization, including all-cause death, recurrent myocardial infarction, and target lesion revascularization	Period of hospitalisation after revascularisation	Clinical observation and recording
Secondary outcomes	Incidence of 1-year MACE, including all-cause death, recurrent myocardial infarction, and target lesion revascularization	1 year after revascularization	Clinical observation and recording
Secondary outcomes	Assessment of quality of life improvement using SAQ	Period of outpatient review after discharge	Scoring with the validated Chinese version of the SAQ
Secondary outcomes	Inflammatory factors, angiogenesis-related factors, exercise-related factors	1 month, 3 months, 6 months, and 12 months after revascularization	Biochemical tests: IL-1, IL-6, and TNF; VEGF, Ang, NO, and eNOS; FSTL1 and FGF 21
Safety outcomes	Monitoring of liver and kidney function, metabolism, and nutrition-related indicators to assess the safety of long-term use of SBP and exercise rehabilitation training	Period of outpatient review after discharge	Biochemical tests: ALT; Scr; ALB; FBG, and HbA1c; TC

MBF, myocardial blood flow; SDS, summed difference score; MACE, major adverse cardiovascular events; SAQ, Seattle angina questionnaire, SBP, Shexiang Baoxin pill, IL-l, interleukin-1, IL-6, Interleukin-6; TNF, tumor necrosis factor; VEGF, vascular endothelial growth factor; Ang, angiopoietin; NO, nitric oxide (NO); eNOS, endothelial nitric oxide synthase, FSTL1, Follistatin-like protein 1; FGF 21, fibroblast growth factor 21; ALT, alanine transaminase; Scr, serum creatinine; ALB, albumin; FBG, fasting blood glucose; HbA1c, glycated hemoglobin A1c; TC, total cholesterol.

### 2.5 Quality control system for the research

To maintain scientific rigor, patient safety, and compliance, this study includes several quality control measures, as detailed below:

#### 2.5.1 Patient compliance management

Patient compliance with SBP and exercise plans will be monitored via a follow-up platform and WeChat group, involving clinicians, rehabilitation physicians, patients, and families. Patients are required to upload daily exercise data. Nurses will communicate in-person with those who miss one or more times of follow-ups. Compliance is defined as logging in more than 3 days a week. The staffing arrangements for routine monitoring and follow-up are shown in Table 8.

**Table 8:** Regular follow-up and monitoring team.

Personnel category	Qualification requirements	Core responsibilities
Rehabilitation physician	Possess cardiovascular rehabilitation qualification	a. Develop personalized plans: determine exercise intensity (low/medium/high), exercise type (Baduanjin, brisk walking, etc.) and progress based on CPET; b. Provide technical guidance: responsible for on-site technical demonstration of Phase I rehabilitation and solving medical problems during exercise; c. Supervise safety: evaluate patients’ IMR based on rechecked CPET results every quarter to adjust exercise plans
Full-time research nurse	Hold cardiovascular specialist nurse certificate	a. Phase I rehabilitation: under the guidance of rehabilitation physicians, assist in on-site supervision of daily rehabilitation training; b. Remote follow-up: responsible for regular follow-up of patients 1–12 months after revascularization, including weekly telephone follow-up, receiving patients’ exercise data, and feeding back abnormal data to rehabilitation physicians; c. Compliance management: conduct targeted communication via phone calls, WeChat groups, etc. with patients who fail to complete exercise as required or miss data submission, analyze the reasons and provide solutions; d. Medication reminder: send medication reminders to patients through the research-WeChat groups at fixed times (7:00 a.m., 12:00 p.m., 7:00 p.m.) daily, and record whether patients take the medicine on time. e. Adherence intervention: for patients with poor medication adherence (such as missing doses frequently), communicate with them to find out the reasons (like forgetting, worrying about side effects) and provide targeted solutions.
Rehabilitation therapist	Possess practical qualification in exercise rehabilitation	a. Phase II rehabilitation: correct exercise postures, guide flexibility training and 4–7-8 breathing technique; b. Phase III rehabilitation support: record standardized exercise teaching videos and upload them to WeChat groups
Cardiovascular SPECIALIST	–	a. Safety control: complete exercise risk stratification for participants during enrollment and exclude high-risk patients; b. Emergency medical support: responsible for evaluation and handling of adverse events during follow-up; c. Medication education: provide patients with detailed medication education before they start taking SBP, including the dosage, frequency, time of administration, possible side effects, and the importance of adherence
Patient’s family	Voluntarily participate and receive training	a. Assist in supervision: remind patients to complete daily training as scheduled; Remind patients to take SBP on time every day according to the prescribed dosage and frequency; b. Emergency training: receive special training on CPR and be able to initially handle emergencies occurring during home-based exercise; c. Feedback communication: keep in touch with the research team (full-time research nurses) and feedback patients’ medication status (whether they take the medicine as required, any abnormal reactions) in a timely manner

#### 2.5.2 Safety assurance

##### 2.5.2.1 Exercise safety

Rehabilitation physicians will offer online and offline exercise guidance. Before starting, patients will receive a thorough assessment, including medical history, symptoms, physical exams, and tests. Physicians will advise on exercise precautions and joint protection for safety.

##### 2.5.2.2 Drug safety

The long-term safety of SBP will be assessed through the monitoring of hepatic and renal function, metabolic processes, and nutrition-related indicators.

##### 2.5.2.3 Comprehensive safety

Before the study, patients will undergo a thorough assessment of cardiovascular, muscular, psychological, and vascular functions to identify risks. Personalized rehabilitation plans will be created from these evaluations, with strict control over the intensity and progress for high-risk patients. Facilities are equipped with first-aid equipment and medications, and medical staff are skilled in first-aid procedures.

#### 2.5.3 Standardized data collection

Data collectors will undergo professional training on standard procedures. Patient information will be recorded using a standardized spreadsheet for consistency. Blood test data collection will adhere to aseptic principles to avoid errors. An electronic medical record system will enable real-time data input and storage, aiding management and queries. Regular reviews will ensure timely corrections and updates.

#### 2.5.4 Exercise intensity adjustment

Cardiopulmonary exercise tests (CPET) will be administered on a quarterly basis to assess the microcirculation resistance index (IMR). Should the IMR surpass a threshold of 25, the exercise intensity will be adjusted accordingly to accommodate the specific requirements of each patient.

#### 2.5.5 Emergency response plan

Patients will receive an emergency kit with nitroglycerin spray for angina pectoris relief. Family members will be trained in cardiopulmonary resuscitation (CPR) to improve their emergency response skills.

### 2.6 Data monitoring

The Data Monitoring Committee (DMC) is composed of supervisors and statistical professionals, and all raw data will be submitted to the management department. As a low-risk intervention study, participants are not anticipated to be exposed to any harmful risks.

### 2.7 Harm

In low-risk studies, the chance of serious adverse events (SAEs) is very low. Adverse events (AEs) may involve patient complaints. Specialized hospital organizations can handle these issues anytime and anywhere. If a subject experiences a serious adverse event due to the intervention during the trial, free treatment or financial compensation will be provided based on medical assessment.

### 2.8 Auditing

The Ethics Committee convenes regularly. The Project Management Group performs monthly checks and reporting. The Trial Steering Committee holds monthly meetings to oversee trial progress. The DMC conducts data reviews every 3–6 months.

### 2.9 Statistical analysis

In this study, there is currently insufficient reference data based on the primary outcome to support sample size calculations; therefore, pilot studies are being conducted to obtain the key parameters required for the formula:


$$n=\varphi^{2}(\sum S_{i}^{2}/g)/[\sum {\left({\bar{X}}_{i}-\bar{X}\right)}^{2}/(g-1)]$$

(where the formula parameter *g* = 4, representing a total of 4 groups in the study) to estimate the required sample size scientifically and accurately, with the significance level set as two-tailed *α* = 0.05, the statistical power set as 1-*β* = 90% (*β* = 0.1), and after calculating the theoretical sample size for each group using the formula upon completion of the pilot studies, a 20% dropout rate will be considered to adjust the theoretical sample size to obtain the final sample size for each group, with the total sample size of the study being the final sample size of each group multiplied by 4.

Statistical analyses will be conducted using appropriate methodologies based on the data characteristics. Continuous variables will be reported as means ± standard deviations, while categorical variables will be presented as frequencies and percentages. Group differences will be assessed using t-tests, analysis of variance (ANOVA), or chi-square tests, as appropriate. A *p*-value of less than 0.05 will be considered indicative of statistical significance. All statistical analyses will be conducted using software such as SPSSAU.

## 3 Discussion

As recommended in clinical guidelines, the core population indicated for CTO-PCI consists of patients with refractory angina despite optimal medical therapy, or those with extensive myocardial ischemia in the occluded territory (21). These subgroups are more likely to derive substantial survival benefits from the procedure. To optimize the selection of surgical strategies, clinicians often evaluate the characteristics of occluded vessels using the Japanese Chronic Total Occlusion Score (J-CTO) system (22). This scoring system includes four coronary angiography (CAG) indicators: blunt stump, calcification, intralesional bend >45°, and occlusion length ≥20 mm. In addition, the diameter of the occluded vessel is also one of the key factors influencing the success rate of the procedure. The antegrade wiring (AW) technique serves as the most commonly used foundational approach in CTO-PCI, while supplementary techniques such as antegrade dissection reentry (ADR), retrograde wiring (RW), and retrograde dissection reentry (RDR) are often employed as adjunctive methods (23, 24). These complementary strategies aim to maximize the success rate and safety of the procedure.

Patients undergoing CTO-PCI frequently have decreased intravascular pressure and coronary blood flow after the surgery; this causes the blocked vessel to appear to have a negative remodeling change, concurrently causing modifications in distal myocardial microvascular structure and function. These adverse changes interact to ultimately trigger CMVD, seriously impacting the long-term prognosis of patients (25). Consequently, accurate evaluation of coronary microvascular function, it is critical for early diagnosis of cardiovascular diseases, optimization of treatment strategies, and prediction of cardiovascular event risk. Cardiovascular magnetic resonance (CMR) imaging, covering coronary flow reserve (CFR) and index of microcirculatory resistance (IMR), widely accepted as a CMVD diagnostic criterion, is a highly promising non-invasive imaging modality of choice (26). Nevertheless, it does not display the real-time dynamic process of myocardial perfusion, and there are strict criteria for inspection equipment, so it might be challenging to perform bedside examinations on individuals who are very sick and immobile. Additionally, despite the fact that CMR can evaluate coronary microvascular function, when it comes to presenting outcomes, it lacks sensitivity and intuition. In this study, myocardial contrast echocardiography (MCE) and stress myocardial perfusion imaging (MPI) will be used as the primary outcomes to assess microvascular improvement. In recent years, these two technologies have gained prominence. Compared with conventional imaging, it provides a data-driven demonstration of myocardial blood perfusion; it also enables a more intuitive assessment of myocardial microcirculatory perfusion status, to provide a fresh method to quantify the functional state of the microvasculature accurately (27–29). Additionally, they have a close association with common outcomes like MACE; it offers a crucial basis for risk prediction and prognostic evaluation of cardiovascular disease (30). By using these technologies, physicians will formulate more accurate treatment plans to advance in cardiovascular disease diagnosis and therapy.

Shexiang Baoxin pill, as a TCM formulation, has been studied for its effects on CMVD. They may act through multiple mechanisms such as promoting angiogenesis, improving endothelial function, and reducing inflammation. SBP has a long-standing history in the treatment of cardiovascular diseases. Derived from traditional Chinese medicine, it has been broadly applied in East Asia for decades to treat conditions like coronary heart disease, heart failure, and hypertension (31). Clinically, a multicenter, double-blind, placebo-controlled phase IV randomized clinical trial involving 2,674 patients with stable coronary artery disease found that SBP, as an add-on to optimal medical therapy, was safe and significantly reduced angina frequency, with a trend toward reduced major adverse cardiovascular events (32). Additionally, clinical practice has shown that combination therapy has a synergistic effect. In the study of heart failure patients with preserved ejection fraction, 60 patients were randomly divided into three groups: group A received SBP combined with home-based exercise training, group B received conventional drugs combined with home-based exercise training, and group C received conventional drug treatment only. After the 12-week intervention, both groups A and B showed significant improvements in peak oxygen uptake, anaerobic threshold, 6-min walking test, Pittsburgh Sleep Quality Index, and SF-36 questionnaire results compared with pre-treatment (*p* < 0.01). Moreover, the improvement degree of group A in some indicators was superior to that of group B, which further demonstrated the positive impact of the combined treatment of SBP and exercise on the patients’ condition (33). These studies have gradually elucidated the role of SBP in cardiovascular diseases and laid the foundation for its clinical application.

In this study, considering the requirements of the patient’s physical condition and long-term compliance, etc., according to the latest cardiac rehabilitation guidelines, exercise rehabilitation is divided into two parts: indoor and outdoor (34). Outdoor exercise using brisk walking: this approach is simple and easy; it has been shown to have a beneficial effect on the rehabilitation of elderly cardiac patients (35). Indoor exercise with Baduanjin, as a low-to-medium-intensity aerobic exercise, because of its site independence and cost-effectiveness, is suitable as a long-term rehabilitation program for our study. Moreover, we will grade patients’ activity intensity based on CPET (36). Based on baseline conditions like the patient’s level of living and the complexity of the disease, etc., develop individualized exercise programs and implement the full-spatial rehabilitation cycle. In-hospital, post-discharge outpatient, and long-term out-of-hospital three-phase, accurately match the frequency and timing of exercises. Following the principle of tertiary rehabilitation in the full cycle of the disease, improve the quality of rehabilitation progressively. In addition, this study followed an approach of the full cycle of participants, a rehabilitation team consisting of cardiologists, rehabilitation therapists, specialized cardiothoracic nurses, carers of patients, etc., supporting patients in multiple dimensions, to ensure the effectiveness of long-term interventions (Figure 3 for details). Exercise rehabilitation is defined by the European Heart Association and the American Heart Association as a class IA treatment for coronary artery disease (37), with high treatment efficacy and cost-effectiveness for improving long-term prognosis (38). The study shows, regular exercise reduces the risk of all-cause mortality by 52%, and the risk of major adverse cardiovascular events by 41% (39).

SBP and exercise rehabilitation interact across multiple physiological action pathways; they all exert positive effects on cardiac function, vascular endothelial function, and hemodynamics. Pharmacokinetic aspects: low-to-moderate-intensity exercise enhances whole-body metabolic status, making the environment conducive to drug metabolism. Particularly in the digestive system, compared to intense exercise, low- to moderate-intensity exercise is beneficial for drug absorption; it allows for faster efficacy and a better degree of absorption (40). Moreover, long-term exercise training also increases the activity of liver enzymes, such as cytochrome P450, which break down medications, lowering the risk of drug buildup in the body (41). The study noted that co-administration of these has a significant impact on enhancing cardiac function and improving the quality of life in patients with cardiovascular diseases (33).

This study has the following advantages: in study design, strict adherence to evidence-based medical norms. The generation of random sequences by computer, and complete distribution concealment with opaque envelope seals, raises the credibility of the study findings. In addition to the primary outcome, the Seattle Angina Questionnaire (SAQ) will also be used to evaluate the quality of life of the patients, to comprehensively reflect the impact of the treatment, on the overall state of the patient from the patient’s subjective feelings, to provide a more thorough viewpoint for evaluating the treatment effect. In addition to routine clinical outcomes, this study will also gather multidimensional data on inflammatory factors, angiogenesis-related and exercise-related factors, digging into the mechanism in detail at the molecular level. Nevertheless, this study also has some limitations. The study was conducted in only one tertiary A hospital; all subjects were recruited from a single source, which may affect the stability and extrapolation of the findings. Moreover, data collection during the trial was made more challenging by the 1-year follow-up period and possible high dropout rate, which will make the study less effective.

In previous cardiac rehabilitation studies, CTO-PCI patients were often overlooked, and it has a high incidence of microvascular dysfunction after surgery and a poor prognosis (4). This study abandons the single-therapy approach of the previous one, using the combined drug and exercise treatment strategies; the aim was to investigate the effect of SBP combined with exercise rehabilitation training on the long-term prognosis of CTO-PCI patients.

After the trial ends, the results will be published in a Chinese or English journal to ensure wide dissemination.

## 4 Conclusion

This protocol presents a methodologically robust and innovative approach to addressing post-CTO-PCI microvascular dysfunction through integrative medicine. By combining SBP’s multi-target pharmacology with precision-guided exercise rehabilitation, the study aims to establish a new paradigm for microvascular protection. Its findings may catalyze future research on synergistic therapies and inform global guidelines for high-risk coronary artery disease populations.
