# Supplementary material for: Effects of Shexiang Baoxin pill combined with exercise rehabilitation training on long-term prognosis of CTO-PCI patients: study protocol for randomized controlled trial
Source: Front Med (Lausanne). 2025 Oct 23;12:1668432. doi: 10.3389/fmed.2025.1668432 (PMC12588978; doi:10.3389/fmed.2025.1668432)
Supplement: Supplementary file 2 [file Table_2.docx]

**No. 1 Baseline Data**

| **Category** | **Specific Items** | **Content to Fill** |
| --- | --- | --- |
| Basic Information | Name Abbreviation | ______ |
|  | Gender | □Male £Female |
|  | Age (years) | ______ |
|  | ID Number (Encrypted) | ______ |
|  | Contact Phone Number | ______ |
|  | Enrollment Date | ______year ______month ______day; |
| Medical History | Date of CTO Diagnosis | ______year ______month ______day; |
|  | Date of PCI Surgery | ______year ______month ______day; |
|  | Past Medical History (Multiple choices allowed) | □Hypertension; £Diabetes; £Hyperlipidemia;  £Myocardial Infarction; £Stroke; £Others ______ |
|  | Family History (Cardiovascular Diseases) | □Yes £No, Details ______ |
| Medication History | Current Medication (Name, Dosage, Frequency) | ______ |
|  | Drug Allergy History | □Yes (Details ______) £No |
| Physical Examination | Height (cm) | ______ |
|  | Weight (kg) | ______ |
|  | BMI (kg/m²) | ______ |
|  | Waist Circumference (cm) | ______ |
|  | Resting Blood Pressure (mmHg) | Systolic ______, Diastolic ______ |
| Laboratory Tests | Fasting Venous Blood Biochemical Indicators | Blood Glucose ______mmol/L,  Total Cholesterol ______mmol/L,  Triglycerides ______mmol/L,  Low-Density Lipoprotein ______mmol/L,  High-Density Lipoprotein ______mmol/L |
|  | Liver and Kidney Function | ALT ______U/L, Scr ______μmol/L, ALB ______g/L |
| Cardiac Function Assessment | Left Ventricular Ejection Fraction (LVEF) | ______% |
|  | New York Heart Association (NYHA) Functional Class | □Class Ⅰ £Class Ⅱ £Class Ⅲ £Class Ⅳ |

| **Category** | **Specific Items** | **Content to Fill** |
| --- | --- | --- |

| Coronary Artery Related | CTO Lesion Vessel | □Left Anterior Descending Artery;  £Left Circumflex Artery; £Right Coronary Artery; £Others ______ |
| --- | --- | --- |
|  | Japanese Chronic Total Occlusion Score (J-CTO) | ______points |
| Others | Availability of Basic Physiological Indicator Measuring Devices (e.g., Electronic Sphygmomanometer) | □Yes £No |
|  | Informed Consent Form Signed | □Yes £No |

**No 2. Study Process Data**

| **Time Point** | **Assessment Items** | **Specific Content** |
| --- | --- | --- |
| Weekly  (Remote Monitoring) | Medication Adherence | □On time (______times/week) □Not on time (Reason: ______) |
|  | Exercise Status | Type: ______, Duration: ______minutes/session,  Frequency: ______sessions/week |
|  | Physiological Indicators | Heart Rate: ______beats/min,  Blood Pressure:_____/_____mmHg |
| Monthly  (Outpatient Follow-up) | Physical Examination | Weight: ______kg,  Blood Pressure:_____/_____mmHg |
|  | Adverse Drug Reactions | □None □Yes (Details: ______) |
|  | Adverse Exercise Reactions | □None □Yes (Details: ______) |
| Every 3 Months | Cardiopulmonary Exercise Test (CPET) | Peak Oxygen Uptake (VO₂peak): ______ml/(kg·min),  Anaerobic Threshold (AT): ______ml/(kg·min) |
|  | Biological Indicators | IL-1: ______pg/ml, IL-6: ______pg/ml, TNF: ______pg/ml, VEGF: ______pg/ml, Ang: ______ng/ml, NO: ______μmol/L, eNOS: ______U/ml, FSTL1: ______ng/ml, FGF21: ______pg/ml |
|  | Liver and Kidney Function | ALT: ______U/L, Scr: ______μmol/L, ALB: ______g/L |
|  | Metabolic Indicators | Fasting Blood Glucose (FBG): ______mmol/L, Glycated Hemoglobin A1c (HbA1c): ______%, Total Cholesterol (TC): ______mmol/L |
|  | Psychological Assessment (Hospital Anxiety and Depression Scale, HADS) | Anxiety Score: ______points,  Depression Score: ______points |

**No. 3 Outcome Measures Data**

| **Outcome Type** | **Assessment Time** | **Assessment Items** | **Specific Content** |
| --- | --- | --- | --- |
| Primary Outcome | □Immediately after surgery;  □1 year after surgery | Coronary microcirculation assessment (core indicator) | Myocardial Contrast Echocardiography (MCE): Myocardial Blood Flow (MBF) ______ml/(min·g) |
|  |  |  | Stress Myocardial Perfusion Imaging (MPI): Summed Difference Score (SDS) ______points |
| Secondary Outcomes | □1 month, □3 months,  □6 months, □12 months after surgery | Incidence of angina pectoris | □No attacks; □With attacks (Number of attacks ______times/assessment cycle, Severity: □CCS Class Ⅰ □CCS Class Ⅱ □CCS Class Ⅲ □CCS Class Ⅳ) |
|  | During hospitalization | In-hospital major adverse cardiovascular events (MACE) | □None; □Yes (Specific events: □All-cause death □Recurrent myocardial infarction □Target lesion revascularization, Occurrence time ______year ______month ______day) |
|  | 1 year after surgery | 1-year MACE | □None; □Yes (Specific events: □All-cause death □Recurrent myocardial infarction □Target lesion revascularization, Occurrence time ______year ______month ______day) |

| **Outcome Type** | **Assessment Time** | **Assessment Items** | **Specific Content** |
| --- | --- | --- | --- |

|  | During outpatient review after discharge (according to follow-up plan), details______ | Quality of life improvement assessment  (Seattle Angina Questionnaire, SAQ) | Physical activity limitation ______points (0-100 points);  Angina stability ______points (0-100 points); Angina attack frequency ______points (0-100 points); Treatment satisfaction ______points (0-100 points); Disease cognition ______points (0-100 points) |
| --- | --- | --- | --- |
|  | □1 month, □3 months,  □6 months, □12 months after surgery | Mechanism-related biological indicators-Inflammatory factors | Interleukin-1 (IL-1) ______pg/ml; Interleukin-6 (IL-6) ______pg/ml; Tumor necrosis factor (TNF)  ______pg/ml |
|  |  | Mechanism-related biological indicators-Angiogenesis-related factors | Vascular endothelial growth factor (VEGF) ______pg/ml; Angiopoietin (Ang) ______ng/ml; Nitric oxide (NO) ______μmol/L; Endothelial nitric oxide synthase (eNOS) ______U/ml |
|  |  | Mechanism-related biological indicators-Exercise-related factors | Follistatin-like protein 1 (FSTL1) ______ng/ml;  Fibroblast growth factor 21 (FGF21) ______pg/ml |
| Safety Outcomes | During outpatient review after discharge (according to follow-up plan), details______ | Liver and kidney function monitoring | Alanine transaminase (ALT) ______U/L;  Serum creatinine (Scr) ______μmol/L;  Albumin (ALB) ______g/L |
|  |  | Metabolic and nutritional indicators | Fasting blood glucose (FBG) ______mmol/L; Glycated hemoglobin (HbA1c) ______%;  Total cholesterol (TC) ______mmol/L |
